# Relative risk reduction: Misinformative measure in clinical trials and COVID-19 vaccine efficacy

**DOI:** 10.1016/j.dialog.2022.100074

**Published:** 2022-11-10

**Authors:** Ronald B. Brown

**Affiliations:** School of Public Health Sciences, University of Waterloo, Waterloo, ON N2L 3G1, Canada

**Keywords:** Relative risk reduction, Relative risk, Absolute risk reduction, Number needed to treat, Number needed to vaccinate, COVID-19 vaccines, Vaccine efficacy, Experimental event rate, Control event rate

## Abstract

Treatment and vaccine efficacy in clinical trials are often reported in the media and medical journals as the relative risk reduction. The present article explains why the relative risk reduction is a misinformative measure that promotes disinformation when reporting efficacy in clinical research studies such as randomized controlled trials for COVID-19 vaccines. The relative risk reduction is based on the relative risk, a proportional measure or ratio used in epidemiologic studies to estimate the probability of a disease associated with an exposure. The present article demonstrates how the relative risk reduction and relative risk obscure the magnitude of disease risk reduction in clinical research. The absolute risk reduction is shown to be a more precise and reliable measure of treatment and vaccine efficacy in clinical research studies. The absolute risk reduction reciprocal also measures the number needed to treat or vaccinate, and is a more accurate measure than the relative risk reduction for comparing risk reductions of clinical studies. Additionally, the present article reviews consequences of COVID-19 vaccine efficacy misinformation disseminated through media reports. The article concludes that relative risk reduction should not be used to measure treatment and vaccine efficacy in clinical trials.

**What is new?:**

• Unreliability of relative measures in clinical trials is graphically illustrated, demonstrating constant relative measures as absolute measures change.
• Misuse of relative measures in clinical research is historically linked to misinterpretation of Jerome Cornfield’s advice on measuring causative and associative effects.
• Consequences of disinformation and misinformation related to COVID-19 vaccine efficacy and modern clinical medicine are described.
• The proper use of absolute measures in meta-analyses is explained.

## Highlight

• Unlike relative risk, absolute risk reduction changes with the baseline risk.
• Relative risk estimates probabilities in observational studies.
• Absolute risk measures causality in clinical trials.
• Using relative risk reduction in clinical trials provides misinformation.
• Relative risk reduction should not be used in clinical trials.

## Graphical abstract

**Figure:**
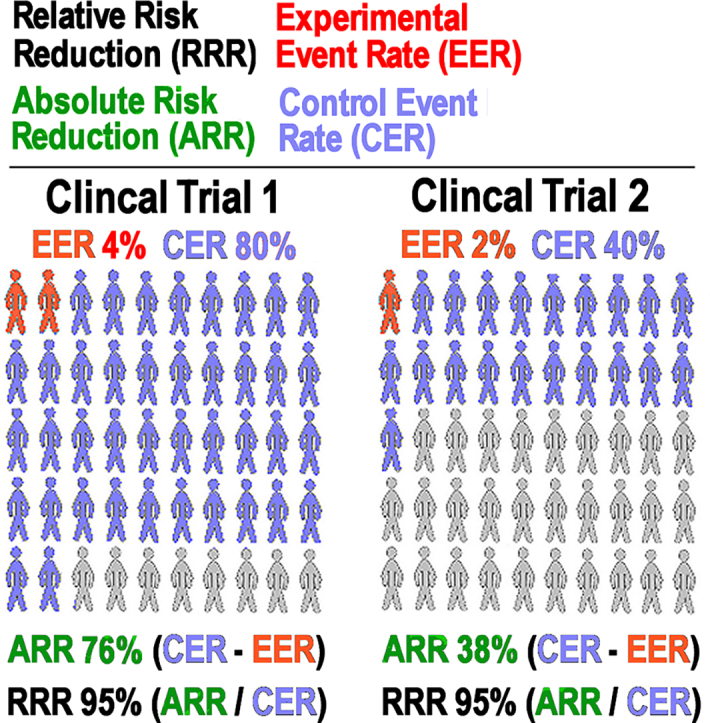

## 1 Introduction

In a vaccine clinical trial for an infectious viral disease like Coronavirus Disease-2019 (COVID-19), participants are randomly assigned to a vaccine group and a placebo group. Vaccine efficacy (VE) is the measure of the vaccine’s ability to prevent an event, a clinical endpoint such as the incidence of an infection, under the controlled experimental conditions of the trial [1]. Randomized controlled trials allow researchers to measure causative relationships between vaccines and clinical endpoints, unlike observational studies of vaccine effectiveness under uncontrolled conditions which cannot measure causation or control all confounding factors.

The traditional method to report vaccine efficacy in the media and medical journals is to use the relative risk reduction (RRR) [2]. Clinical trial risk equations (1-5) are shown in Table 1. A risk is an event rate in a group. The RRR is based on the relative risk (RR), which is a proportional measure or ratio calculated by dividing the event rate in the vaccine or experimental group, the experimental event rate (EER), by the event rate in the placebo or control group, the control event rate (CER) (1). The RRR is calculated by subtracting the RR from 1 (2). The risk difference, also known as the absolute risk reduction (ARR), is calculated by subtracting the experimental event rate from the control event rate (3). An alternative method to calculate the RRR is to compare the ARR relative to the control group that didn’t receive the vaccine. That is, the RRR is calculated by dividing the ARR by the control event rate (4). The control event rate is also known as the baseline risk, so the relative risk reduction is the risk difference relative to the baseline risk. The number needed to treat (NNT), the reciprocal of the ARR, is the number of people who must be treated to prevent one event (5). Numerically, risk can be expressed as a decimal number or as a percentage (%) by moving the decimal point two places to the right (multiplying by 100%).

**Table 1:** Clinical trial risk equations.

1.	Relative Risk	= Experimental Event Rate/Control Event Rate
2.	Relative Risk Reduction	= 1 – Relative Risk
3.	Absolute Risk Reduction	= Control Event Rate – Experimental Event Rate
4.	Relative Risk Reduction	= Absolute Risk Reduction/Control Event Rate
5.	Number Needed to Treat	= 1/ARR

Importantly, critical inconsistencies can develop between the RRR and ARR in a clinical trial if they are not interpreted correctly. Misinterpreting the RRR can cause the reported results to appear very much larger than the ARR, as occurred in the clinical trial reports for the COVID-19 mRNA vaccines [3]. Additionally, because dividing a number by a fraction produces a larger number, dividing the ARR by the placebo infection rate using the alternative calculation method often converts the ARR into a larger RRR.

Current advice on assessing clinical trial results and vaccine efficacy recommends reporting both the ARR and RRR [4]. Nevertheless, continued omission of the ARR in reported results of clinical trials remains a controversial issue. The present article provides an in-depth examination of this issue with several objectives. First, to demonstrate the inconsistent relationship between absolute and relative measures. Second, to argue that relative measures are intended for associative relationships and serve no purpose in randomized controlled trials that measure causative relationships. Finally, the author’s personal point of view offers insights and directions for future research, including a section on consequences of vaccine efficacy misinformation disseminated by media reports.

## 2 ARR Variability and RRR Consistency

Table 2 shows a hypothetical example of several vaccine clinical trial results in which decreases in the ARR are dependent on decreased infection rates in the vaccine and placebo groups— the EER and CER, respectively. Nevertheless, the RR and the RRR in this example remain unchanged. These inconsistencies demonstrate that relative measures cannot be relied on to accurately interpret changes in absolute measures.

At the same RR and RRR, Table 2 shows that the vaccine ARR can vary from a very high 95% rate to a very low rate of 0.95%. Similar to the last row of Table 2, the approximate RR, RRR, and ARR for the Pfizer/BioNTech BNT162b2 and Moderna mRNA-1273 COVID-19 vaccines are shown below [3]:

BNT162b2: 0.04% vaccine infection rate / 0.75% placebo infection rate = 5% RR, 95% RRR, 0.7% ARR

Mrna-1273: 0.07% vaccine infection rate / 1.2% placebo infection rate = 5% RR, 95% RRR, 1.1% ARR

Table 3 shows more examples of changes in the ARR that are dependent on infection rate changes, with consistent RRs and RRRs.

**Fig. 1:**
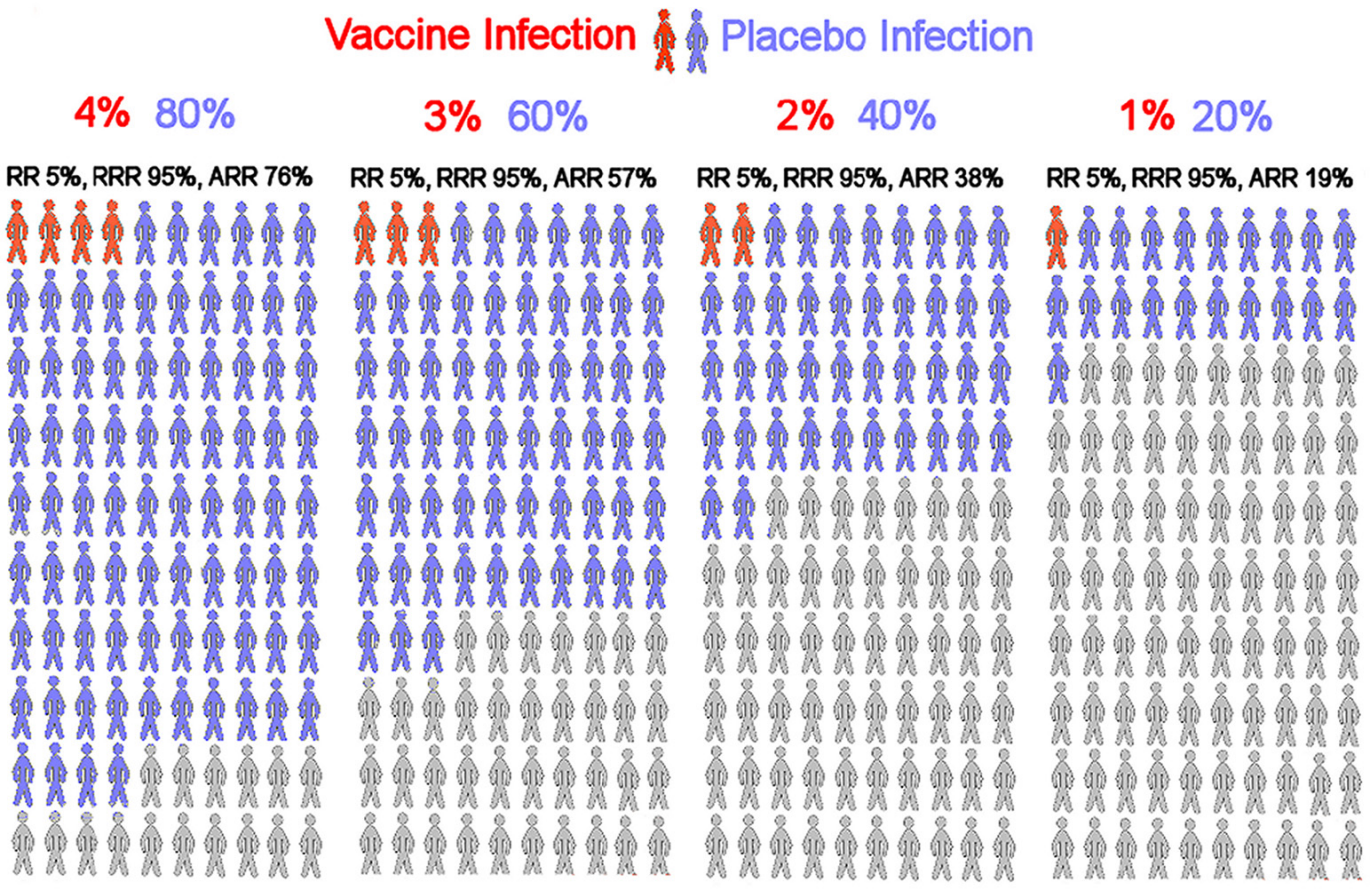
ARR is dependent on infection rates in trials with consistent RRs and RRRs.

Figure 1 is a graphic illustration of four hypothetical vaccine trials demonstrating how changes in the ARR (76%, 57%, 38%, and 19%) are dependent on baseline risk changes of the placebo group—the denominator of the relative risk (80%, 60%, 40%, and 20%). The consistent relative measures in the trials (5% RR and 95% RRR) verify that a clinical treatment’s absolute benefit at a given relative risk “could vary considerably as the baseline risk changes” [5]. Variability of the ARR at a given relative risk explains why the RRR is an unreliable and inconsistent measure to interpret changes in a treatment’s absolute clinical benefits. This raises the question discussed in the next section: what is the purpose of the RRR in reporting results of randomized controlled clinical trials?

## 3 Criticism of relative risk in clinical research

First use of relative measures in clinical research is attributed to the work of biostatistician Jerome Cornfield who conducted research on tobacco and lung cancer in the 1950s at the U. S. National Cancer Institute [6]. Cornfield wrote, "Both the absolute and the relative measures serve a purpose," explaining that a relative measure can be used to appraise "the possible noncausal nature of an agent having an apparent effect," and "the absolute measure would be important in appraising the public health significance of an effect known to be causal" [7].

Accordingly, relative measures are used today in epidemiological studies investigating associations between exposures and risks, such as risk ratios of disease incidence in cohort studies and odds ratios of disease prevalence in case studies and cross-sectional studies [8]. By contrast, absolute measures are used in randomized controlled trials, considered the gold standard of evidence demonstrating cause-effect relationships in clinical treatments and interventions [9].

Implications of Cornfield’s remarks on the proper use of relative and absolute measures seem clear: absolute measures should not be used in observational studies of associative relationships, nor should relative measures be used in clinical trials of causative relationships. For example, when reporting an increased risk in an observational study, one must qualify the increased risk as being *associated* with the exposure, not caused by the exposure. Associations between exposures and disease outcomes in epidemiological studies most often use relative measures, in contrast to public health interventions that measure absolute differences in treatment outcomes [10]. Correspondingly, a vaccine in a clinical trial that reduces the absolute risk under controlled conditions is a causative outcome, and should not be reported using a relative measure. Arguing in favor of absolute outcome measures in clinical trials, Sprenger and Stegenga [11] point out that "relative outcome measures are neither necessary nor sufficient for choosing between two interventions."

### 3.1 ARR Magnitude

Writing in *The Lancet*, authors from the Center for Drug Evaluation and Research of the U.S. Food and Drug Administration (FDA) described the important difference between a decreased risk within a group of people and a relative risk or risk ratio, which is a proportion of risk outcomes in separate groups:

“Relative risk does not measure ‘risk’ at all, because risk has dimensions, such as observed deaths per 100 or 1000 people. However, a risk ratio has no dimensions because they cancel in calculating the ratio” [12].

The authors pointed out that a 50% risk ratio for a drug tested in 100 people that reduces two deaths to one death (0.01 / 0.02 = 0.5) is the same as the risk ratio for a drug tested in a larger group of 1000 people that reduces two deaths to one death (0.001 / 0.002 = 0.5). However, the authors explained that the reductions are not actually a 50% decreased risk. Using absolute measures, the authors showed that “the change from two deaths per 100 people to one death per 100 people is a 1% decreased risk,” (ARR: 0.02 – 0.01 = 0.01 * 100 = 1%). “And the change from two deaths per 1000 people to one death per 1000 people is a 0.1% decreased risk,” (ARR: 0.002 – 0.001 = 0.001 * 100 = 0.1%), which is ten times smaller in magnitude than the decreased risk per 100 people.

ARR measures the precise magnitude and strength of the reduced risk, essential for clinical evaluation, which the RRR obscures [13]. Yet, ARRs “tend to be ignored because they give a much less impressive effect size than RRRs" [14]. Nevertheless, “clinical research has a substantial need for absolute measures,” and researchers have argued that “the RR should no longer be used in clinical trials” [15].

## 4 Disinformation in disseminating COVID-19 vaccine efficacy

Disinformation is defined as “false information that’s spread with the specific intent of misleading or deceiving people” [16]. The FDA published guidelines for communicating risks and benefits from research studies, which state that both the RRR and ARR should be reported to the public [4]. Although RRRs were reported in the media and scientific journals by vaccine manufacturers and the FDA Advisory Committee that authorized and approved the COVID-19 mRNA vaccines, ARRs were not reported, denying the public important information needed before consenting to vaccination.

**Fig. 2:**
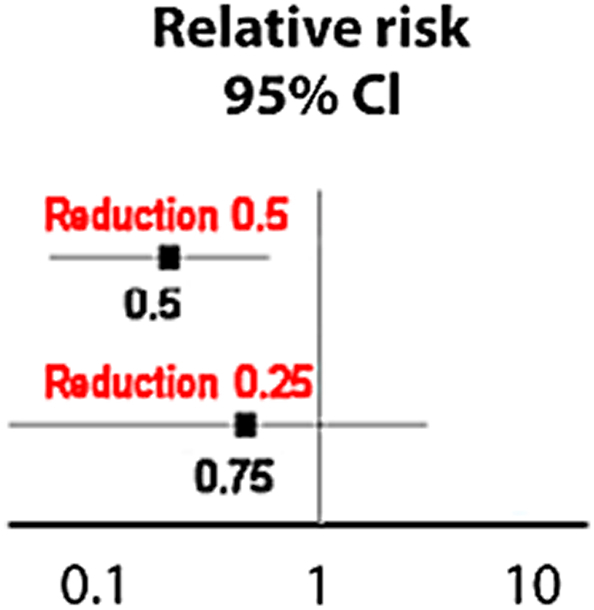
Meta-analysis comparing relative risks (black) and relative risk reductions (red) on a logarithmic scale.

As demonstrated in Table 2 and Table 3, reporting the RRR in a clinical trial designed to measure causation under experimental conditions can be misleading. Additionally, the public is often encouraged to believe that a vaccine with reported 95% efficacy means that 95% of vaccinated people will be protected. This is incorrect [17]. Vaccine efficacy doesn’t report a risk reduction. Rather, VE reports a *relative risk* reduction calculated by subtracting the relative risk from the null value of 1. The null value is the point estimate of the relative risk with identical risks in groups exposed and unexposed to a risk factor, i.e., the null value indicates no change in the risk from exposure to a risk factor relative to non-exposure. The relative risk reduction is used in meta-analyses to compare the magnitude and direction of relative risks in observational studies [18], as shown in Fig. 2.

**Table 2:** ARR and infection rate changes at 5% RR and 95% RRR.

Vaccine (EER)	Placebo (CER)	RR	RRR	ARR
5%	100%	5%	95%	95%
4%	80%	5%	95%	76%
3%	60%	5%	95%	57%
2%	40%	5%	95%	38%
1%	20%	5%	95%	19%
0.5%	10%	5%	95%	9.5%
0.05%	1%	5%	95%	0.95%

**Table 3:** ARR and infection rate changes with consistent RRs and RRRs.

Vaccine (EER)	Placebo (CER)	RR	RRR	ARR
10%	100%	10%	90%	90%
8%	80%	10%	90%	72%
6%	60%	10%	90%	54%
4%	40%	10%	90%	36%
2%	20%	10%	90%	18%
25%	100%	25%	75%	75%
20%	80%	25%	75%	60%
15%	60%	25%	75%	45%
10%	40%	25%	75%	30%
5%	20%	25%	75%	15%

Forest plots in Fig. 2, the horizontal lines, represent the RR 95% confidence intervals (CI) in a meta-analysis [19]. If the confidence interval includes the null value of 1, the relative risk is considered statistically non-significant. Fig. 2 calculates the 0.5 and 0.25 relative risk reductions (red) by subtracting the respective 0.5 and 0.75 relative risks (black) from 1. The correct use of the relative risk reduction in meta-analyses of observational studies is very different from the absolute risk reduction in controlled experimental studies or randomized clinical trials.

As described in equation 4 of Table 1, the VE or relative risk reduction can be calculated by dividing the ARR by the control event rate or baseline risk. Based on this equation, some authors claim that the RRR in a randomized controlled trial compares the reduced risk of a treatment relative to the placebo group that didn’t receive the treatment [20], but this claim is flawed. The reduced risk of a treatment, the ARR, is calculated by subtracting the treatment group risk from the placebo group risk. Comparing the ARR relative to the placebo group risk only serves to mathematically divide the ARR by a fraction and inflate the ARR into a higher RRR. On the other hand, the ARR in equation 4 of Table 1 can be rewritten as equaling the RRR multiplied by the baseline risk. Accordingly, if the RRR remains constant, the ARR changes as the baseline risk changes, demonstrating yet again that the ARR can change independently of the RRR.

Without reporting the ARR and correcting the public’s misunderstanding of vaccine efficacy, dissemination of vaccine efficacy as the RRR is meaningless and misleading disinformation. Framing vaccine efficacy as a relative measure can lead to cognitive bias that overestimates outcome effects [11].

“The relative risk does not provide any information about the absolute risk of the event occurring, but rather the higher or lower likelihood of the event in the exposure versus the non-exposure group” [21].

Furthermore, eliminating the RRR from clinical trials will prevent intentionally reporting the RRR without the ARR—thus helping to reduce treatment and vaccine efficacy disinformation that is prevalent in biomedical research [22,23]. Examples of biomedical disinformation do not appear to be innocent mistakes or oversights—they could be evidence of deliberate industry misconduct and corruption that warrants further investigation.

Perhaps the most devastating of all disinformation consequences due to the misuse of relative measures in reporting clinical research occurs as the integrity of systematic reviews and clinical trial meta-analyses is undermined. Subsequently, clinical practice guidelines that unintentionally rely upon biased clinical research to treat patients safely and effectively is also undermined. These clinical consequences of relative risk disinformation could account for much of the reason why error in medical treatment is the third leading cause of U.S. deaths [24], and why healthcare in the United States is among the least effective systems among high-income nations [25]. The following section explains the proper use of absolute measures in meta-analyses.

### 4.1 ARR and NNT in meta-analyses

The *Cochrane Handbook for Systematic Reviews of Interventions* states that relative measures provide greater consistency than absolute measures when pooling together results from clinical trials in meta-analyses [26]. Yet, the reciprocal of the ARR, the number needed to treat (NNT) or vaccinate (NNV) to reduce one clinical event, is a more precise measure of treatment strength than relative measures.

“It is a common perception that consistency of an effect measure in meta-analysis means that it is the effect measure of choice. This again is a misconception because if the RR does not measure effect magnitude and simply reflects the prevalence of the outcome in the study, consistency is no longer meaningful” [15].

On the other hand, unlike risk ratios which “may be fairly independent of the patients’ baseline risk status,” ARR and NNT measures can be pooled together in meta-analyses if “the event rates in the control groups are very similar” [27]. Alternatively, ARR and NNT measures in analyses of several trials with dissimilar baseline risks should be reported individually.

"When numbers needed to treat are presented for an intervention, the setting in which it occurred, the time period, the outcome, and the baseline risk of the patients for whom the number needed to treat is thought to be applicable should be described" [28].

Furthermore, dissimilar baseline risks in control groups of a treatment may indicate a need to examine clinical trial data for biases, confounding factors, and effect modifiers [29], which relative measures may overlook.

## 5 Consequences of COVID-19 vaccine efficacy misinformation

Misinformation is defined as “false information that is spread, regardless of whether there is intent to mislead” [16]. One of the consequences of misinforming the public that the COVID-19 mRNA vaccine efficacy is very high is the expectation that the vaccines will protect people from severe infections, hospitalizations, and death [30]. This expectation is reinforced by observational studies showing that vaccinated people are more likely to have mild and moderate infections. But such observations only provide evidence of associations and are not causative evidence that the vaccines protect against severe infections, hospitalizations, and death.

The primary endpoints of the COVID-19 mRNA vaccine clinical trials were a non-severe infection with at least one clinical symptom [31,32]. Furthermore, “the mRNA vaccine clinical trials were not powered to address severe disease” [33], nor were the trials “designed to detect a reduction in any serious outcome such as hospital admissions, use of intensive care, or deaths” [34]. Additionally, healthy people were enrolled in the clinical trials—but in order to test the clinical endpoints of severe infections, hospitalizations, and death, participants selected for a clinical trial should have a higher risk of these conditions than healthy people. Randomized clinical trials “often include patients with a high prior risk for the outcome of interest” [35].

### 5.1 Breakthrough infections and vaccine hesitancy

In April 2021, U.S. Centers for Disease Control and Prevention (CDC) issued a notice that breakthrough infections of COVID-19 in vaccinated individuals would no longer be recorded unless the cases were hospitalized [36]. The CDC policy appears to assume that the reported high efficacy of the vaccines from the clinical trials would be sufficient to prevent most mild and moderate infections in vaccinated people, allowing CDC resources to concentrate on surveillance of more serious conditions.

Additionally, with very low efficacy in the COVID-19 vaccines, alternative factors could explain high rates of hospitalized COVID-19 cases in unvaccinated people [30]. One plausible explanation is that vaccine hesitancy most often occurs in minority groups like Blacks and Hispanics [37]. These groups also have higher rates of obesity, poor nutritional status, and chronic disease, which lower immune protection and increase susceptibility to infection [38]. People with low socioeconomic status are also more likely to delay treatment and rely on emergency-room services rather than visit private offices of primary healthcare providers [39].

### 5.2 Vaccine immunogenicity

Claims have been published in the media and scientific journals that vaccine effectiveness has waned and that booster injections are needed [40]. But observational evidence of vaccine effectiveness within a population overlooks the vaccines’ low efficacy in clinical trials, which calls into question the validity of the vaccines’ immunogenic mechanism. To stimulate immune protection against COVID-19, the genomic sequence of an injected particle of synthetic messenger ribonucleic acid (mRNA) is proposed to initiate ribosomal translation of the Severe Acute Respiratory Syndrome-Coronavirus-2 (SARS-CoV-2) spike S protein *in vivo* [41]. Furthermore, encapsulation within a lipid nanoparticle is intended to protect injected mRNA from destructive defense mechanisms within the cell cytoplasm [42]. Yet, the fate of encapsulated mRNA is not clear. As extracellular vesicles surround the lipid nanoparticle, synthetic mRNA may be isolated and destroyed by lysosomal degradation enzymes [43]. Additionally, translation of any synthetic mRNA released into the cytoplasm may be blocked by micro ribonucleic acid (microRNA) [44].

### 5.3 Vaccine mandates

Finally, heads of governments, schools, healthcare facilities, and private businesses, misled by the vaccines’ reported 95% RRR, enforced vaccine mandates upon workers and the public who use their services [45]. The result is that some workers resigned from their jobs, students dropped out of school, and many people were coerced into accepting an unwanted medical procedure under the threat of job or salary loss. Evidence shows that U.S. public health departments have resorted to similar employee and employer coercion in campaigns to increase vaccination rates for smallpox as far back as the early 20^th^ century [46]. Additionally, World Health Organization has not supported vaccine mandates previous to the enforcement of COVID-19 vaccine mandates [47], and legal challenges against the mandates have grown [48].

## 6 Conclusions

Key issues regarding use of relative measures in clinical trials and vaccine efficacy are summarized as follows:

1. At any relative risk in a clinical trial, which measures the proportion or ratio of the experimental and control event rates, variability of the absolute risk reduction is dependent on changes in the baseline risk of the control group.
2. Relative risk and relative risk reduction measures are more suitable for observational studies that estimate probability of an exposure associated with a risk, while absolute risk reduction is more reliable for reporting risk reductions causatively related to the efficacy of a vaccine or treatment in a randomized controlled trial.
3. Absolute risk reduction measures and the number of individuals needed to be treated or vaccinated to reduce one event should not be pooled together in meta-analyses unless the baseline risks are similar.
4. Misusing the relative risk reduction to report treatment and vaccine efficacies of clinical trials leads to scientific disinformation and media misinformation, especially if the absolute risk reduction is not also reported.
5. For the reasons stated above, relative risk and relative risk reduction are misinformative measures of treatment and vaccine efficacy and should not be used in randomized clinical trials.

## Funding

This research received no external funding.

## Institutional review board statement

Not applicable.

## Informed consent statement

Not applicable.

## Data availability

Data for Pfzier/BioNTech BNT162b2: https://doi.org/10.1056/nejmoa2034577; data for Moderna mRNA-1273: https://doi.org/10.1056/NEJMoa2035389 (accessed on 10 January 2021).

## Declaration of Competing Interest

The author declares no conflict of interest.

## References

[1] cdc.gov (December 4, 2021). Principles of epidemiology in public health practice, lesson 3: Measures of risk. Section 6: Measures of public health impact 2012. https://www.cdc.gov/csels/dsepd/ss1978/lesson3/section6.html.

[2] Dasgupta S. (2019). A review of vaccine efficacy measures. Vaccin Res Open J.

[3] Brown R.B. (2021). Outcome reporting bias in COVID-19 mRNA vaccine clinical trials. Medicina..

[4] Fischhoff B., Brewer N., Downs J. (2011).

[5] Cook R.J., Sackett D.L. (1995). The number needed to treat: a clinically useful measure of treatment effect. Bmj..

[6] Schlesselman J.J. (2016 Jan). Jerome Cornfield’s Bayesian approach to assessing interim results in clinical trials. J R Soc Med.

[7] Cornfield J., Haenszel W., Hammond E.C. (1959). Smoking and lung cancer: recent evidence and a discussion of some questions. J Natl Cancer Inst.

[8] cdc.gov (December 6, 2021). Principles of epidemiology in public health practice, lesson 1: Introduction to epidemiology. Section 7: Analytic Epidemiology 2012. https://www.cdc.gov/csels/dsepd/ss1978/lesson1/section7.html.

[9] Hariton E., Locascio J.J. (2018 Dec). Randomised controlled trials - the gold standard for effectiveness research: Study design: randomised controlled trials. Bjog..

[10] Szklo M., Nieto F.J. (2014).

[11] Sprenger J., Stegenga J. (2017). Three arguments for absolute outcome measures. Philos Sci.

[12] Stadel B.V., Colman E., Sahlroot T. (2005 Apr 9-15). Misleading use of risk ratios. Lancet.

[13] Noordzij M., van Diepen M., Caskey F.C. (2017). Relative risk versus absolute risk: one cannot be interpreted without the other. Nephrol Dial Transplant.

[14] Olliaro P., Torreele E., Vaillant M. (2021). COVID-19 vaccine efficacy and effectiveness—the elephant (not) in the room. Lancet Microbe.

[15] Doi S.A., Furuya-Kanamori L., Xu C. (2020 Nov 7). Controversy and Debate: Questionable utility of the relative risk in clinical research: Paper 1: A call for change to practice. J Clin Epidemiol.

[16] dictionary.com (November 3, 2021). Disinformation vs. misinformation 2021. https://www.dictionary.com/browse/disinformation.

[17] Olliaro P. (2021). What does 95% COVID-19 vaccine efficacy really mean?. Lancet Infect Dis.

[18] Schünemann H., GE V, JP H, et al (February 2021). Chapter 15: Interpreting results and drawing conclusions. https://training.cochrane.org/handbook/current/chapter-15.

[19] Lewis S., Clarke M. (2001). Forest plots: trying to see the wood and the trees. BMJ..

[20] Irwig L., Irwig J., Revena L. (2008).

[21] Tenny S., Hoffman M.R. (2020).

[22] Sismondo S. (2021). Epistemic Corruption, the Pharmaceutical Industry, and the Body of Medical Science. Front Res Metr Anal.

[23] Morreim E.H. (2021 Jan-Jun). Corporations, high-stakes biomedical research, and research misconduct: yes they can (and sometimes do). J Law Biosci.

[24] Makary M.A., Daniel M. (2016). Medical error—the third leading cause of death in the US. BMJ..

[25] Tikkanen R., Abrams M.K. (March 7, 2022). US health care from a global perspective, 2019: higher spending, worse outcomes 2020. https://www.commonwealthfund.org/publications/issue-briefs/2020/jan/us-health-care-global-perspective-2019.

[26] Deeks J.J., Higgins J.P., Altman D.G. (2019).

[27] Cates C.J. (2002). Simpson’s paradox and calculation of number needed to treat from meta-analysis. BMC Med Res Methodol.

[28] Smeeth L., Haines A., Ebrahim S. (1999). Numbers needed to treat derived from meta-analyses—sometimes informative, usually misleading. BMJ..

[29] online.stat.psu.edu (December 6, 2021). Epidemiological research methods, 3.5 - bias, confounding and effect modification 2021. https://online.stat.psu.edu/stat507/lesson/3/3.5.

[30] Griffin J.B., Haddix M., Danza P. (2021). SARS-CoV-2 infections and hospitalizations among persons aged≥ 16 years, by vaccination status—Los Angeles County, California, May 1–July 25, 2021. Morb Mortal Wkly Rep.

[31] Baden L.R., El Sahly H.M., Essink B. (February 4, 2021). Efficacy and safety of the mRNA-1273 SARS-CoV-2 vaccine. N Engl J Med.

[32] Polack F.P., Thomas S.J., Kitchin N. (2020 December 16). Safety and efficacy of the BNT162b2 mRNA covid-19 vaccine. N Engl J Med.

[33] Tenforde M.W., Self W.H., Adams K. (2021). Association between mRNA vaccination and COVID-19 hospitalization and disease severity. JAMA..

[34] Doshi P. (2020). Will covid-19 vaccines save lives? Current trials aren’t designed to tell us. BMJ..

[35] Bruynesteyn K., Wanders A., Landewé R. (2004). How the type of risk reduction influences required sample sizes in randomised clinical trials. Ann Rheum Dis.

[36] Birhane M., Bressler S., Chang G. (2021). COVID-19 vaccine breakthrough infections reported to CDC—United States, January 1–April 30, 2021. Morb Mortal Wkly Rep.

[37] Quinn S.C., Andrasik M.P. (2021). Addressing vaccine hesitancy in BIPOC communities — Toward trustworthiness, partnership, and reciprocity. N Engl J Med.

[38] Stefan N., Birkenfeld A.L., Schulze M.B. (2021). Global pandemics interconnected — obesity, impaired metabolic health and COVID-19. Nat Rev Endocrinol.

[39] Kangovi S., Barg F.K., Carter T., Long J.A., Shannon R., Grande D. (2013). Understanding why patients of low socioeconomic status prefer hospitals over ambulatory care. Health Aff.

[40] Dolgin E. (2021). COVID vaccine immunity is waning-how much does that matter?. Nature..

[41] Kwon D. (December 23, 2020). The Promise of mRNA Vaccines November 25, 2020. https://www.the-scientist.com/news-opinion/the-promise-of-mrna-vaccines-68202.

[42] Schlich M., Palomba R., Costabile G. (2021 Mar 20). Cytosolic delivery of nucleic acids: The case of ionizable lipid nanoparticles. Bioeng Transl Med.

[43] Behzadi S., Serpooshan V., Tao W. (2017 Jul 17). Cellular uptake of nanoparticles: journey inside the cell. Chem Soc Rev.

[44] O’Brien J., Hayder H., Zayed Y. (2018). Overview of MicroRNA biogenesis, mechanisms of actions, and circulation [Review]. Front Endocrinol.

[45] Gostin L.O., Salmon D.A., Larson H.J. (2021). Mandating COVID-19 vaccines. JAMA..

[46] Koehler J.P. (1925). How Milwaukee aborted its smallpox epidemic. Wis. Med. J..

[47] WHO (2021).

[48] Rothstein M.A., Parmet W.E., Reiss D.R. (2021). Employer-mandated vaccination for COVID-19. Am J Public Health.

